# Set Shifting Training with Categorization Tasks

**DOI:** 10.1371/journal.pone.0081693

**Published:** 2013-12-04

**Authors:** Anna Soveri, Otto Waris, Matti Laine

**Affiliations:** Department of Psychology and Logopedics, Abo Akademi University, Turku, Finland; University of Texas at Dallas, United States of America

## Abstract

The very few cognitive training studies targeting an important executive function, set shifting, have reported performance improvements that also generalized to untrained tasks. The present randomized controlled trial extends set shifting training research by comparing previously used cued training with uncued training. A computerized adaptation of the Wisconsin Card Sorting Test was utilized as the training task in a pretest-posttest experimental design involving three groups of university students. One group received uncued training (n = 14), another received cued training (n = 14) and the control group (n = 14) only participated in pre- and posttests. The uncued training group showed posttraining performance increases on their training task, but neither training group showed statistically significant transfer effects. Nevertheless, comparison of effect sizes for transfer effects indicated that our results did not differ significantly from the previous studies. Our results suggest that the cognitive effects of computerized set shifting training are mostly task-specific, and would preclude any robust generalization effects with this training.

## Introduction

Following an increased awareness of the magnitude of brain plasticity [1], the trainability of cognitive functions and the possible generalization (transfer effects) of training to other untrained functions has received much interest during the last years [2]–[4]. The trainability of executive functions has stirred particular interest, because they represent general high-level control functions that are involved in a great number of mental activities and thus of great importance to cognitive performances. Furthermore, the clinical potential of executive training and rehabilitation is considerable given the occurrence of executive dysfunction in a wide range of conditions [5].

From the training and rehabilitation perspective, it is usually not interesting to improve cognitive performance on the trained task per se, but to improve performance on other untrained tasks, elicit general improvement in related skills, and ultimately increase the quality of everyday life. This encompasses the idea of *transfer*. The possible generalization of learning can involve *near transfer*, in other words, transfer between similar contexts, and *far transfer*, which refers to transfer between contexts [6]. Here, near transfer is defined as generalization within the same cognitive domain, and far transfer as generalization to another cognitive domain, due to some shared underlying cognitive-neural components in the tasks. Despite a number of studies investigating training of different executive functions, there is, however, still little consensus regarding transfer effects as a result of executive training, as studies have reported near transfer, far transfer, both near and far transfer, and no transfer at all. Several studies have found that training effects are, in fact, limited only to the trained task and its close variants [1], [7].

A number of theories have addressed the organization of executive functions [8]–[10]. Often postulated subcomponents of executive functions include inhibition, planning, set shifting, and working memory updating [11]–[12]. The present study will focus on training of set shifting and its possible transfer effects to untrained tasks. Set shifting (or task switching) refers to the ability to switch between tasks or mental sets, and this ability is considered to be highly relevant for everyday life [13]. With regard to possible transfer of set shifting training, near transfer to structurally similar but untrained set shifting tasks would speak for a shared (and malleable) cognitive basis that emerges despite task-specific features. In the same vein, far transfer would require that there are at least some shared cognitive-neural components between seemingly different tasks. Unfortunately, earlier training studies have not put forth theoretical models that would detail these shared components. However, studies that have found significant correlations between set shifting, inhibition, and working memory updating [9], [ see 14 for associations between set shifting and inhibition], as well as between set shifting and intelligence measures [15]–[17], [ but see 18], indicate commonalities between these domains. At a more theoretical level, recent developmental evidence [19] suggests that flexible task switching is related to the use of active abstract representations. This enables generalization of rules to novel stimuli, an important feature in fluid intelligence tasks such as the Raven's Progressive Matrices.

In experimental research, set shifting is typically measured by simple alternating tasks such as adding 2 to a given number vs. subtracting 2 from a given number [13], [20]. Two different blocks of trials are usually created, namely *single-task* blocks and *mixed-task* blocks. The former ones consist of *single-task* trials with only one set of task rules, and no set shifting is required. In the mixed-task blocks, however, the tasks are alternated either predictably or unpredictably, creating *repetition* trials (non-shifting trials) and *switching* trials. One can then study two types of processing costs by comparing performance on repetition trials vs. switching trials, on the one hand, and on single-task trials vs. repetition trials, on the other. The performance difference between switching trials and repetition trials is coined as the *switching cost* [13], which can be seen as a measure of task-set reconfiguration [21], interference from the previous task-set [22], or a combination of both [13], [23]. The performance difference between single-task trials and repetition trials, on the other hand, is labeled as the *mixing cost* [13], and can be considered as a measure of the extra processing required for maintaining more than one set of task rules [24].

Only a few studies have examined training of set shifting and its possible transfer effects. Minear and Shah [25] conducted three experiments in which they studied the near transfer effects of set shifting training with categorization tasks in healthy young adults. They employed cued (the correct response was clearly indicated by the stimuli) set shifting training with predictable stimulus sequences (a task switch occurred on every third trial) vs. unpredictable stimulus sequences (participants had no way of predicting an incoming task switch) in three training tasks. Possible generalization was measured with a structurally similar but untrained near transfer task. The results showed decreased mixing and switching costs for both training groups on the training tasks. However, transfer was evident only in the mixing cost in the transfer task with unpredictable switching in the group that trained with unpredictable stimulus sequences. Their second experiment was identical to the first one, except that for both training and transfer tasks, the cue and the task to be performed were separated by a 200 ms interval. This manipulation was devised for two purposes. Firstly, it would verify that the first finding was not peculiar to rapid switching. Secondly, it should ameliorate possible differences in task-set bias between predictable and unpredictable switching. In other words, participants might show greater commitment to the current task-set if they can rely on getting advance notice of the next trial type. The results were almost identical to those of the first experiment. In the final experiment, the authors had an additional untrained cue type in the near transfer tasks to study if participants were learning cue-specific strategies. The transfer effect was again observable, indicating that the training was not the result of cue-specific learning.

In another set shifting training study, Karbach and Kray [26] examined the training effects in three different age groups (children, young adults, and older adults). They also studied the effects of verbal self-instruction and varied set shifting training by changes in tasks and stimuli during training. The shifting of the task set was fully predictable, i.e., the participants were instructed to switch on every second trial in the mixed-task blocks. The training results showed near transfer to both the switching and mixing cost in a structurally similar, untrained set shifting task. Children and older adults showed a more pronounced transfer effect to the mixing cost than young adults did. Verbal self-instruction did not increase transfer effects, while variation in training interacted with age so that adults benefited more from varied training than children did. Remarkably, the results showed far transfer to an inhibition task, a verbal and a spatial working memory task, and to a fluid intelligence task. The authors suggested that the far transfer they observed is due to demands set by the training task on other executive processes, such as inhibition.

The two previous studies investigating training of set shifting [25], [26] employed either cued training or a fully predictable task sequence. In the present experiment, we studied for the first time whether training with uncued, unpredictable task sequences would affect training gains in set shifting. We did this by comparing the training effects of cued vs. uncued unpredictable shifting. The training task was a categorization task based on the Wisconsin Card Sorting Test (WCST), a complex executive task that taps particularly set shifting [9], but also inhibition [27] and working memory [28], as well as other cognitive processes such as abstract reasoning, strategic planning, problem-solving, and organized search [29]. There were two crucial differences between the cued and the uncued task version in the present study. In the uncued version, the participant had no information as to when a change in the sorting rule would take place and what the new sorting rule would be. In the cued version, on the other hand, the participant was informed of both the oncoming shift in set and the new sorting rule. Several studies have indicated that different cognitive processes are in use when set shifting is predictable compared to when it is not. For example, a functional magnetic resonance imaging (fMRI) study [28] comparing brain activation in healthy adults performing three different variants of the WCST (card sorting with unpredictable uncued, predictable uncued, and predictable cued set shifting), revealed increased prefrontal activation for the unpredictable and uncued version, compared to the other variants. The authors suggested that this is related to the increased demands on working memory and attentional control when set shifting is both unpredictable and uncued [28]. An event-related potential study with healthy adults [30] indicated that different mechanisms are involved in the shifting process when the shift in set is externally driven (cued) and when it is endogenously generated (uncued). It takes several trials to recover from an unpredictable and uncued shift, possibly due to the need to strengthen the representation of the new set in working memory and inhibit the interference from the previous set. The idea that different cognitive processes are involved in predictable and unpredictable set shifting has also found support in clinical studies. For example, patients with focal frontal lesions have been shown to perform better on the WCST when they are aware that a shift in sorting rules will take place [31].

In order to also explore possible transfer effects to untrained tasks, we included five transfer tasks tapping four cognitive domains: set shifting, inhibition, working memory, and general intelligence. Following the promising results of training set shifting [25]–[26] and other executive functions [32]–[36], transfer effects could be expected. At the same time, it has been claimed that transfer can occur only between cognitive functions that at least partially share the same brain regions [37] and engage the same cognitive processes [35]. Thus, we hypothesized that the most probable outcome of our training would be near transfer to a related untrained set shifting task, evidenced by decreased cognitive costs for set shifting. Furthermore, we speculated that the cognitively more demanding practice of self-generated set shifting in the uncued training task version could elicit more pronounced training gains than the cued training task version. Predictions about far transfer were more difficult to make. However, based on previous research suggesting that unpredictable and uncued card sorting engages working memory and inhibition to a greater extent than predictable and cued shifting does, we hypothesized that if card sorting training can lead to far transfer, it is more likely to be a result of training with uncued rather than cued shifting.

## Materials and Methods

### Ethics Statement

The study had been approved by the Institutional Review Board of the Department of Psychology and Logopedics, Abo Akademi University, and all participants gave their written informed consent.

### Participants

The participants consisted of 42 right-handed, healthy Finnish university students in the age-range of 20–30 years (*M* = 23.43, *SD* = 2.33), of which 20 were male and 22 were female (Table 1). The participants were matched within triads according to their pretest uncued switching cost and based on this, randomly allotted into three groups: (a) the uncued training group, (b) the cued training group, and (c) the control group. Each group consisted of 14 participants and there were no significant differences between the three groups regarding age or the WAIS-III [38] Similarities score, *F*s <1. The three groups were also balanced regarding gender, χ^2^(2, *N* = 42) = 1.34, *p* = .513. All participants received a reimbursement of 50 €.

**Table 1:** Summary of Demographics and Average Performance on WAIS-III Similarities.

	Uncued training	Cued Training	Control
n	14	14	14
Age in years (SD)	23.43 (2.85)	23.71 (2.30)	23.14 (1.88)
Sex M/F	5/9	8/6	7/7
WAIS-III Similarities (SD)	22.43 (2.98)	23.36 (2.31)	23.08 (2.90)

### Procedure

All participants took part in the pre- and posttest sessions. During the pretest session, they were asked to give their written informed consent and fill in a Finnish translation of the Edinburgh Handedness Inventory [39] as well as a background information sheet probing their date of birth, education, occupation, vision, hearing, possible reading difficulties, possible neurological and psychiatric illnesses, medication, subjective level of alertness, and possible alcohol intake during the 24-hour period preceding the testing. In addition to these background questionnaires, the pretest consisted of the WAIS-III Similarities and the following six computerized tasks: WAIS-III Digit span, Raven's Standard Progressive Matrices (Raven's SPM [40]), the categorization task based on the WCST, the Number-Letter task (set shifting [41; adapted from 21]), the Simon task (inhibition of task-irrelevant information [42]), and an N-back task with digits (working memory updating [43]). A more detailed description of each test can be found below.

After the pretest, the training groups practiced with the cued or the uncued version of the categorization task for two weeks, three times a week. Each training session lasted for approximately thirty minutes. The control group did not receive any training.

The posttest session was administered within one week after completed training. This session consisted of the same tasks that were administered at the pretest, with the exception of the Similarities. The pre- and posttest sessions, as well as the training sessions, were administered in groups with a maximum of three participants per group.

### The categorization task

In this task, four different stimulus cards appeared in a horizontal line at the top of the computer screen. The task was to match response cards, appearing one at a time, with the stimulus cards, based on different sorting rules.

**Figure 1:**
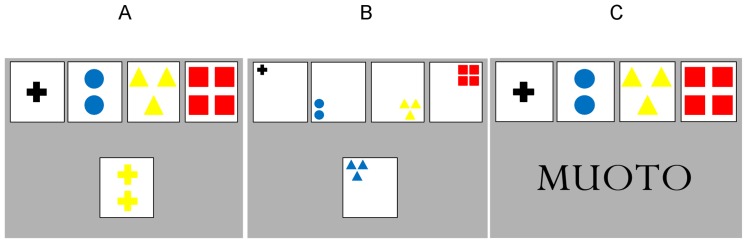
The two test versions of the categorization task. (A). The easier test version of the task. The stimulus cards are at the top of the screen and the response cards appear in the center of the bottom half of the screen. If the correct sorting rule is color, the subject should press button “3”. (B). The difficult version of the task. The shape size has been decreased to enable the positioning of the figures. If the correct sorting rule is location, the subject should press button “1”. (C). Training with cued presentation of the stimuli. The written cue was presented at the beginning of each set. MUOTO is the Finnish word for “shape”.

The task employed two difficulty levels. In the easier version (Figure 1A), the four stimulus cards were composed of different shapes (cross, circle, triangle, or square), colors (red, blue, yellow, or black), and quantities (one, two, three, or four figures). The task was to sort the response cards according to these features, thus yielding three possible sorting rules: *shape*, *color*, and *quantity*. Sorting was to be done by deciding which stimulus card had figures of the same shape, color, or number, as the figures on the response cards, based on the sorting rule valid at that moment. In the difficult version of the task (Figure 1B), *location* (upper left, upper right, lower left, or lower right corner) was added as a fourth sorting rule and feature on the stimulus cards.

In the easier version of the categorization task, the sorting rule changed in a random fashion after five to ten response cards, and in the more difficult version after six to ten, regardless of whether the responses were correct or incorrect. The set length of five was removed from the difficult version in order to decrease the possibility that the participants would not have had time to discover the sorting rule before it changed.

Within each difficulty level, two separate versions were created with cued vs. uncued presentation of the stimuli. In the *cued* version (Figure 1C), each time the sorting rule was about to change, the new correct sorting rule was given in written form on the computer screen (SHAPE, COLOR, QUANTITY or LOCATION, written in Finnish). In the *uncued* version, no cues of the sorting rules were given, meaning that the participant had to come up with the correct sorting rule solely on the basis of the feedback given by the computer after each response. The feedback was given audio-visually: a correct response elicited a high pitch tone and a bright screen, while an incorrect response or no response elicited a low pitch tone and a dark screen. Feedback was given in all task versions.

At the beginning of the task, only the four stimulus cards were shown on the screen for 1000 ms. In the uncued version, this was followed by the first response card in the middle of the lower part of the screen. The response card remained on the screen until a response key was pressed or until 3000 ms had passed. Before moving on to the next response card, audio-visual feedback was given for 1500 ms. The cued version was identical to the uncued version, except that the verbal cue was presented for 1000 ms at the beginning of each set, after the stimulus cards had appeared. The cue was not present during the presentation of the response cards. There were four response keys covered with stickers carrying the numbers 1, 2, 3, 4 that represented the four stimulus cards, numbered from left to right.

#### The categorization task at the pre- and posttest

Both the cued and the uncued version of the categorization task were included also in the pre- and posttests, to evaluate the participants' improvement on the criterion task itself. At the pre- and posttest, the cued single-task blocks (i.e., no change in sorting rule, see the Introduction for a description) of each sorting rule, the mixed-task (i.e., alternation between sorting rules) cued version, and the mixed-task uncued version were administered to the participants. To acquaint the participants with the categorization principles, they were first administered the four cued single-task blocks with 20 trials each. In order to avoid switching in the single-task blocks, each block was started separately. With regard to the cued and uncued mixed-task versions of the categorization task, only the difficult versions were used in the pre- and posttest due to their higher processing demands and thus greater sensitivity in detecting change. Both mixed-task versions consisted of 160 trials (one of each set length for each sorting rule), each version including 20 switching trials. The mixed-task versions were preceded by a short practice sequence including all sorting rules.

The response cards were ordered manually to guarantee that a card following a switch of sorting rule could not fulfill the criteria for both the previous and current rule. The order of the sorting rules was pseudorandomized separately for the cued and uncued versions.

The dependent measures on both versions of the categorization task were the mixing cost and the switching cost in reaction times (RTs). The mixing cost was calculated as the difference between the repetition-trial mean from the mixed-task block and the single-task mean from the single-task blocks. The switching cost in RTs was the repetition-trial mean subtracted from the switching-trial mean from the mixed-task block. The switching- and repetition-trial means were derived separately from the cued and the uncued version. The single-task mean was calculated by averaging the RTs across the four single-task blocks. In the cued version, the mean for the switching trials was calculated by averaging the RTs on the first trial of each set, as this was the trial following a change in sorting rule. In the uncued version, on the other hand, task feedback served as the cue that a task switch had taken place. Thus the mean for the switching trials was calculated by averaging the RTs on the second trial in each set. In the cued version, the non-switching repetition-trial mean was calculated by averaging the RTs of each trial except the first one of every set that was the switching trial. In the uncued version, the non-switching repetition-trial mean included the RTs of each correct response following a correct response. For example, if the sequence of responses were correct - correct - correct - incorrect, the second and third correct answer were included in the repetition-trial mean.

In addition to RTs, incorrect responses in the uncued categorization task were also analyzed. Because incorrect responses are inherent in the nature of the uncued version, simply counting incorrect responses is not an adequate measure as many of the responses are valid guesses (attempts to find the new sorting rule). An error was therefore defined as an incorrect response of which the participant should have been aware of, based on the feedback they had received. For example, if a participant received feedback that a response including both shape and color criteria was incorrect but nevertheless tried to sort according to shape or color during the same set, the response was scored as an error. Missed responses were also counted as errors. Only RTs were analyzed in the cued version, due to a very low number of incorrect responses.

#### The categorization task in training

The cued training group practiced with the cued version and the uncued training group with the uncued version of the categorization task. During the first week the participants trained with the easier task version, and during the second week with the difficult version.

The easier version of the task included 540 trials (four of each set length for each sorting rule), thus yielding a total of 72 switching trials, and the difficult version 640 trials (four of each set length for each sorting rule), resulting in 80 switching trials. The trials were divided into six four-minute blocks. A 20-second pause was placed in-between blocks, during which the word PAUSE written in Finnish was shown on the display. Participants could opt for a longer pause on completing the first three training blocks.

The order of the sorting rules was pseudorandomized, while the response cards were randomized separately for each participant, with the restriction that no response card was identical to the stimulus cards. Task performance during training was not included in the statistical analyses.

### Transfer tasks

#### The Number-Letter Task

The Number-letter task [41; adapted from 21] was utilized in this study as a near transfer measure to set shifting. In this task, number-letter pairs were presented in the middle of the screen in one of two squares, one square being above the other. If the number-letter pair was presented in the upper square, the participant had to determine if the number was even or odd by pressing one of the two response keys on the computer keyboard. If the number-letter pair was presented in the lower square, the participant had to determine if the letter was a vowel or a consonant. The location of the number-letter pair, thus, served as a cue for which task to perform.

The trials were divided into switching trials in which the location of the number-letter pair had changed, and repetition trials in which the number-letter pair was in the same location as in the previous trial. Three trial blocks were created: two single-task blocks and one mixed-task block. In the first single-task block of 32 trials, the number-letter pairs were always presented in the upper square. In the second single-task block of 32 trials, the number-letter pairs were always presented in the lower square. There was an equal amount of even and odd trials in block 1 and vowel and consonant trials in block 2. The mixed-task block consisted of 80 trials, of which 32 were switching trials and 48 repetition trials. The switching trials were divided into an equal amount of number-to-letter switches and letter-to-number switches. Also the 48 repetition trials were divided into an equal amount of number and letter decisions. Each block was preceded by a practice sequence of 16 trials.

Every trial began with a blank screen. After 150 ms, a fixation cross appeared in the middle of the screen. The fixation cross was replaced by two squares (one of which contained a number-letter pair) after 300 ms. The squares remained on the screen until a response had been given or until 3000 ms had passed. Two response keys were used in this task, one for vowels and even numbers, and the other for consonants and odd numbers.

The dependent measures on this task were the mixing cost (the difference between repetition trials and single-task trials averaged over block 1 and 2) and the switching cost (the difference between switching trials and repetition trials) in RTs and in the proportions of errors.

#### The Simon Task

The Simon task [42] was used to measure possible far transfer to the ability to inhibit task-irrelevant information. In the present task version, a blue or red square was presented on the left or the right side of the computer screen. The participant had to press the left key if the square was blue and the right key if the square was red, irrespective of the location of the square. The trials were either congruent or incongruent. On congruent trials, the stimulus location was on the same side as the response key (e.g., a blue square on the left side of the screen) and on incongruent trials, on the opposite side (e.g., a red square on the left side of the screen).

A total of 100 trials were administered, of which half were congruent and half incongruent. The order of the trials was randomized separately for each participant. The trials were divided into four equally long blocks with a five-second break in-between. Before starting the actual task, every subject received a practice sequence.

A fixation cross was presented at the beginning of each trial. The cross disappeared after 800 ms and was replaced by a blank screen for 250 ms. After this, a blue or red square was presented on either the left or right side of the screen. The stimulus remained on the screen until a response key was pressed or until 1000 ms had passed. Then the screen went blank for 500 ms before moving on to the next trial.

The dependent variables on this task were the Simon effect in RTs and proportions of errors. The Simon effect is the difference between incongruent and congruent trials, and taps the processing cost related to the incompatible location of the stimulus.

#### Digit span

The Digit span test from the WAIS-III was included to measure possible far transfer to working memory and attention. In this test, the task was to repeat sequences of digits (sequence length: 2–9 digits) in the same and in reversed order as they were presented. In the present computerized version, a fixation cross was presented for 1000 ms in the middle of the screen at the beginning of each digit sequence. After this, the screen went blank for 1000 ms, and then the first number was presented in the middle of the screen. After 1000 ms the screen went blank again for 1000 ms before the next number appeared. The text “Type your answer” appeared in Finnish at the end of each sequence. There was no time limit for typing in the response.

#### The N-back Task

The N-back task [43] was used to measure possible far transfer to the ability to update information in working memory. In this task, numbers from 1 to 9 were presented one at a time at the center of the screen and the task was to remember the previous number (1-back) or the one presented three trials back (3-back). The total amount of trials in this task was 240, of which 120 were 1-back trials and 120 3-back trials. The trials were divided into 12 blocks (six 1-back blocks and six 3-back blocks) of 20 trials. Each block consisted of 9 targets and 11 non-targets (altogether 54 targets and 66 non-targets). The presentation order of the stimuli was pseudorandomized.

Two response keys were used. The participant was to press the right key each time the trial was a target, i.e., the number was the same as the previous number (1-back) or the one three trials back (3-back), and the left key each time the trial was a non-target, i.e., the number did not match.

Before each block, a written prompt informing the participant of whether the following block was a 1-back or a 3-back block, appeared on the screen together with a picture of a hand that indicated the corresponding response keys. After 5000 ms, the first number became visible and remained on the screen for 1500 ms. After this, the number was replaced by a fixation cross with a duration of 450 ms. The fixation cross was then followed by the next number. On each trial, the response had to be given within 2000 ms. As it was not possible to give an answer on the first trial in the 1-back condition and on the first three trials in the 3-back condition, these trials were excluded from the analysis.

The difference in RTs and error rates (in percent) between the 1-back and the 3-back conditions were used as the dependent variables for this task. These N-back effect measures reflect the processing cost caused by the increased demands on working memory updating in the 3-back condition.

#### Raven's SPM

The computerized Raven's SPM used in this study consisted of sets “B”, “C”, “D” and “E” [40]. These sets were divided into two equal parts (part 1 and 2) with 24 trials each. Subjects were randomly assigned to be pretested with either part 1 or part 2. Three practice trials were administered at the beginning of the task. The task ended either upon completion or when participants had worked on the task for 20 minutes.

### Statistical analyses

#### The categorization tasks

ANCOVAs with posttest performance as the dependent variable, and pretest performance as a covariate [44]–[45], were run on the switching cost in RT (difference between switching and repetition trials) in the cued and the uncued task version, on the mixing cost (difference between repetition and single-task trials) in RT in the uncued task version, and on the total number of errors in the uncued task version that provided enough data for analysis. One should note here that the categorization tasks showed reliable switching and mixing costs except for the cued categorization task where all groups showed negative mixing costs at pretest (Table 2). These negative mixing costs are most likely related to a task order effect where the single-task blocks were presented first, leading to learning that then facilitated performance in the cued mixed-task block. Accordingly, the mixing cost for the cued categorization task was not included in the subsequent analyses.

**Table 2:** Mean Response Latencies (in milliseconds) with Standard Deviations in the Cued Categorization Task.

			Uncued Training		Cued Training		Control	
			(n = 13)		(n = 14)		(n = 14)	
		Time	*M*	*SD*	*M*	*SD*	*M*	*SD*
Reaction times	Switching trials	Pretest	1084	222	1034	225	1028	245
		Posttest	774	150	754	130	905	190
	Repetition trials	Pretest	800	166	815	163	796	166
		Posttest	612	85	630	92	728	100
	Single-task trials	Pretest	816	137	846	177	808	138
		Posttest	622	110	609	81	709	114
	Mixing cost	Pretest	−16	149	−30	56	−12	89
		Posttest	−10	41	21	41	18	70
	Switching cost	Pretest	284	143	219	85	232	163
		Posttest	162	114	124	70	177	126

As regards the between-subjects factor, we ran separate ANCOVAs comparing the cued group to the control group, the uncued group to the control group, and the cued group to the uncued group.

#### Transfer tasks

For the transfer tasks, similar ANCOVAs with pretest performance as a covariate, posttest performance as the dependent variable, and group as a between-subjects factor were conducted for each transfer task. In the Number-letter task, ANCOVAs were performed on the switching and mixing cost in RTs and error rates, in the Simon task on the Simon effect in RTs, in the N-back task on the N-back effect in RTs and error rates, in the Digit span forward and backward on span length, and in the Raven's SPM on correct responses. Furthermore, in the analysis of the Raven's SPM, test part (first half; second half) was included as a within-subjects factor. Two orthogonal contrasts were defined for the between-subjects factor Group (cued; uncued; control). The first contrast compared the two training groups to the control group (−1 −1 2) and the second contrast the two training groups to each other (1 −1 0). The analyses were conducted only on RTs for the Simon task, due to very low error rates.

Each task was reviewed independently regarding possible exclusion of individual cases. In all tests, the exclusion criteria were being an extreme outlier in error rates/test scores at pretest and/or showing evidence of misunderstanding test instructions. In the interest of space, for all analyses on the transfer tasks, the reporting of the results will be restricted to significant main effects of group.

## Results

### Categorization tasks

One subject belonging to the uncued training group was removed from the statistical analyses on the categorization task for being an extreme outlier on the pretest error rates in the uncued version.

#### Cued training group vs controls

The ANCOVA on the switching cost in RTs in the trained, cued task (Table 2) showed no significant main effect of group, *F*(1, 25) = 1.80, *p* = .191, η^2^_partial_ = .067. The main effect of group was non-significant also in the analyses on the switching and the mixing cost in RTs in the untrained, uncued task (Table 3), *F*s <1. The results from the ANCOVA on errors in the uncued task, however, showed a nearly significant main effect of group, *F*(1, 25) = 3.84, *p* = .061, η^2^_partial_ = .133, stemming from a lower number of incorrect responses in the cued group at posttest.

**Table 3:** Mean Response Latencies (in milliseconds) and Incorrect Responses with Standard Deviations in the Uncued Categorization Task.

			Uncued Training		Cued Training		Control	
			(n = 13)		(n = 14)		(n = 14)	
		Time	*M*	*SD*	*M*	*SD*	*M*	*SD*
Reaction times	Switching trials	Pretest	1358	241	1323	260	1414	337
		Posttest	1074	249	1159	226	1385	248
	Repetition trials	Pretest	876	138	888	165	924	156
		Posttest	656	103	684	92	861	162
	Mixing cost	Pretest	60	141	42	106	116	73
		Posttest	34	59	76	68	152	181
	Switching cost	Pretest	482	224	435	204	490	237
		Posttest	419	190	474	202	524	194
Incorrect responses		Pretest	19.00	7.93	20.29	7.14	19.29	6.03
		Posttest	4.77	3.68	9.07	5.47	12.93	5.03

#### Uncued training group vs controls

The ANCOVA on the switching cost in RTs in the trained, uncued task failed to show a significant main effect of group, *F*(1, 24) = 2.14, *p* = .156, η^2^_partial_ = .082, although there was a decrease in the switching cost in the uncued group and an increase in the control group. The ANCOVA on the mixing cost in RTs in the uncued task, however, showed a main effect of group that approached significance, *F*(1, 24) = 3.36, *p* = .079, η^2^_partial_ = .123, stemming from a smaller mixing cost in the uncued group than the control group (for which the mixing cost increased from pretest to posttest). Furthermore, the ANCOVA on errors in the uncued task showed a significant main effect of group, *F*(1, 24) = 21.01, *p*<.001, η^2^_partial_ = .477, indicating that fewer errors were made by the uncued group than the control group at posttest. The ANCOVA on the switching cost in RTs in the untrained, cued categorization task showed no significant differences between groups, *F*<1.

#### Cued training vs uncued training groups

The ANCOVA on the switching cost in RTs in the cued task showed no group difference, *F*<1. The ANCOVA on the mixing cost in RTs in the uncued task, however, showed a difference between the two training groups that approached significance, *F*(1, 24) = 3.15, *p* = .088, η^2^_partial_ = .116, stemming from a lower mixing cost in the uncued group than the cued group (for which the mixing cost increased from pretest to posttest). In the ANCOVA on error rates in the uncued task, there was a significant main effect of group, *F*(1, 24) = 5.20, *p*<.05, η^2^_partial_ = .178, due to the fact that the uncued group made less errors than the cued group after training. The analysis on the switching cost in RTs in the uncued task, showed no significant main effect of group, *F*<1.

### Transfer tasks

Two subjects belonging to the cued training group and one subject belonging to the uncued training group were removed from the Number-Letter statistical analyses for being extreme outliers in error rates at pretest. One subject from the uncued training group was removed from the analysis on the Digit Span Backward for misunderstanding the instructions. Regarding the N-back task, seven participants misunderstood the instructions and two participants were excluded due to being outliers on their error rates. Technical complexities of the computerized N-back task combined with simultaneous testing of multiple participants contributed to the unusually high subject loss. Four of the excluded subjects belonged to the uncued training group, two belonged to the cued training group and three belonged to the control group. All participants were included in the statistical analyses of the Raven's SPM and the Simon task.

The results of the ANCOVAs on performance on the Number-letter task (Table 4), the Simon task (Table 5), the Digit span (Table 6), and the Raven's SPM (Table 7) revealed no significant main effects of group. The ANCOVA on the N-back effect in error rates in the N-back task (Table 8), however, showed a significant main effect of group, *F*(2, 29) = 4.39, *p* = .022, η^2^_partial_ = .232. The planned contrasts revealed that the N-back effect in error rates was significantly larger in the training groups than in the control group at posttest (*p* = .016). The second contrast comparing the training groups with each other was not significant.

**Table 4:** Mean Response Latencies (in Milliseconds) and Error rates (in Percent) with Standard Deviations in the Number-Letter Task.

			Uncued Training		Cued Training		Control	
			(n = 13)		(n = 12)		(n = 14)	
		Time	*M*	*SD*	*M*	*SD*	*M*	*SD*
Reaction times	Switching trials	Pretest	1067	157	1259	217	1038	184
		Posttest	933	203	1058	213	919	155
	Repetition trials	Pretest	800	148	921	223	738	148
		Posttest	686	200	750	178	644	84
	Single-task trials	Pretest	651	80	665	174	600	77
		Posttest	600	110	585	79	541	57
	Mixing cost	Pretest	149	112	256	134	138	152
		Posttest	86	117	165	122	103	58
	Switching cost	Pretest	267	148	338	148	300	133
		Posttest	247	105	308	120	275	106
Error rates	Switching trials	Pretest	2.88	2.00	2.60	1.80	2.23	2.27
		Posttest	1.44	1.62	2.60	2.61	2.01	3.15
	Repetition trials	Pretest	0.80	1.05	0.69	1.03	0.74	1.32
		Posttest	1.12	1.08	0.69	1.36	0.15	0.56
	Single-task trials	Pretest	1.44	1.35	0.78	1.05	1.00	0.99
		Posttest	1.56	2.21	1.17	0.97	1.45	1.43
	Mixing cost	Pretest	−0.64	1.28	−0.09	1.25	−0.26	1.15
		Posttest	−0.44	2.23	−0.48	1.67	−1.30	1.35
	Switching cost	Pretest	2.08	2.62	1.91	2.17	1.49	2.11
		Posttest	0.32	2.01	1.91	2.34	1.86	3.30

**Table 5:** Mean Response Latencies (in milliseconds) with Standard Deviations in the Simon Task.

		Uncued Training		Cued Training		Control	
		(n = 14)		(n = 14)		(n = 14)	
	Time	*M*	*SD*	*M*	*SD*	*M*	*SD*
Congruent trials	Pretest	451	54	469	68	417	52
	Posttest	456	90	449	65	425	39
Incongruent trials	Pretest	473	52	494	68	449	47
	Posttest	465	82	469	52	443	58
Simon effect	Pretest	22	25	25	23	32	23
	Posttest	10	30	19	26	18	26

**Table 6:** Mean Span Length with Standard Deviations in Digit Span Forward and Backward.

		Uncued Training		Cued Training		Control	
		(n = 14/13)		(n = 14)		(n = 14)	
Condition	Time	*M*	*SD*	*M*	*SD*	*M*	*SD*
Forward	Pretest	5.93	0.92	6.36	1.08	6.50	1.09
	Posttest	6.50	1.16	6.57	1.28	6.71	1.14
Backward	Pretest	5.00	1.53	5.86	1.79	6.36	1.22
	Posttest	5.54	1.90	5.86	1.51	6.43	1.34

*Note*. The uncued training group consists of 14 participants on Digit Span Forward and 13 on Digit Span Backward.

**Table 7:** Mean Scores and Standard Deviations in the Raven's SPM.

	Uncued Training		Cued Training		Control	
	(n = 14)		(n = 14)		(n = 14)	
	*M*	*SD*	*M*	*SD*	*M*	*SD*
Pretest	19.43	2.31	19.00	2.75	19.86	3.01
Posttest	20.07	1.54	20.36	2.71	20.43	1.79

*Note*. Scores can range from 0–24.

**Table 8:** Mean Response Latencies (in milliseconds) and Error Rates (in Percent) with Standard Deviations in the N-back Task.

			Uncued Training		Cued Training		Control	
			(n = 10)		(n = 12)		(n = 11)	
		Time	*M*	*SD*	*M*	*SD*	*M*	*SD*
Reaction times	1-back trials	Pretest	594	99	621	93	572	102
		Posttest	542	100	590	101	536	48
	3-back trials	Pretest	871	160	924	118	848	164
		Posttest	764	178	851	121	730	165
	N-back effect	Pretest	277	93	303	80	276	145
		Posttest	222	119	261	116	194	168
Error rates	1-back trials	Pretest	2.89	1.71	2.05	1.81	2.87	2.04
		Posttest	2.28	3.44	1.54	2.51	2.15	2.81
	3-back trials	Pretest	30.69	12.39	18.38	10.73	18.81	13.66
		Posttest	16.57	9.62	14.05	9.43	5.35	6.64
	N-back effect	Pretest	27.79	11.70	16.34	10.91	15.94	13.73
		Posttest	14.29	10.27	12.52	10.46	3.20	7.14

## Discussion

Prior set shifting training studies have indicated that both near [25] and far transfer [26] are achievable through set shifting training. These studies employed only cued or predictable shifting during training. The present study attempted to extend this research by contrasting cued vs. uncued categorization training in order to examine the training gains with externally driven vs. endogenously generated shifts in set [30].

The group comparisons revealed a statistically significant training effect for the uncued training group in the errors elicited by the demanding uncued categorization task. A similar but not quite significant training effect was obtained for the mixing cost in the same training task version. These effects attest to an improvement on the trained task for the uncued training group. Moreover, also the cued training group showed a nearly significant reduction of errors in the uncued categorization task when compared to the controls, suggesting some performance improvement on a non-trained but closely related task.

Regarding transfer effects, no statistically significant near or far transfer was seen to other untrained tasks as a result of training with the categorization task. However, compared to the training groups, the control group showed greater improvement in working memory updating performance over time. This is difficult to interpret, and may represent a chance finding (nine participants had to be excluded from this task analysis).

The present results are thus in line with those studies that show improvement on the trained task and some improvement on a structurally similar task, but limited, if any transfer to tasks that measure other cognitive functions [7], [25], [35], [46]–[48]. The lack of statistically significant transfer effects in our study suggests that the training gains we observed were process-specific. This conforms with the claim that transfer is possible only when the training task and the transfer task engage the same cognitive processes and overlapping neural systems [35], [49].

We expected that the uncued version would result in a better training outcome than the cued version, due to the more demanding endogenous shifting [30] inherent in the uncued task. This was borne out only in the most limited sense of the word, as the statistically significant training effects of the uncued training group only dealt with the task they trained with.

However, there are at least two factors that can limit the statistically significant results from our study. First, there are differences in the exposure to training between studies. While we had a total of 3540 training trials against the 1768 training trials in the Karbach and Kray [26] study which showed both near and far transfer as a result of training, their training setup included more switching trials (884 switching trials against 432 switching trials in our cued and 480 switching trials in our uncued training). Second, due to practical limitations, the group sizes in our study were quite small. This decreases the statistical power and increases the risk for Type II errors in our study. Indeed, sensitivity power analyses (with an α-level of .05 and power of .8) indicate that only large effects, η^2^_partial_ = .198–.232 [as defined by 50], can be detected with the sample sizes used in this study. The effect sizes for the non-significant main effect of group in the near transfer task (Number-letter task), for example, range from η^2^_partial_ = .015 to η^2^_partial_ = .075 (with the best performance in the uncued group). This corresponds to small to medium effects that are not large enough to be detected by the statistical test. Also, although the cued group did not improve significantly more than the control group on the trained task, the effect size for the main effect of group on the switching cost was of medium size (η^2^_partial_ = .067), suggesting that also this non-significant result can be related to our small sample sizes.

Furthermore, we evaluated the heterogeneity of effect sizes in the present study compared to the Minear and Shah [25] and Karbach and Kray [26] studies, by using Hedge's Q test [51]. For the tests of heterogeneity of effect sizes, we chose effects that were as comparable as possible regarding the measures and analyses used. The choice of effects was also related to the availability of statistics in the two previous studies.

Both previous studies found significant transfer effects after set shifting training. The analyses on the effects of training on the mixing cost in the near transfer task showed that there was no significant difference in effect sizes among the present study and the two previous studies, *Q*(2) = 1.374, *p* = .503. There was also no significant difference in effect sizes between the present study and the Karbach and Kray [26] study regarding far transfer to inhibition, *Q*(1) = 0.241, *p* = .624, and fluid intelligence, *Q*(1) = 0,139, *p* = .709. Again, these findings suggest that our small sample sizes limited the statistical results we obtained. The N-back task was not included in the tests of heterogeneity of effect sizes, due to the fact that it was the control group that showed the greatest improvement from pretest to posttest in the present study.

Another possible reason for the fact that the present study did not find near transfer to the untrained set shifting task may lie in the type of near transfer task that we used. Based on previous studies [35], [25], the near transfer task should have close resemblance to the training task for near transfer effects to appear. In other words, the near transfer task should share the cognitive processes (and at least partly the activated brain regions [37]) that are engaged during training. The Number-letter task used in the present study, however, was quite different from the categorization tasks used in training. It is therefore possible that a perceptually more similar Number-Letter task (e.g., with similar shapes and colors as in the categorization task) would provide transfer effects. Furthermore, the set shifting in the Number-letter task was unpredictable and cued, not unpredictable and uncued, as was the case in the uncued version of the categorization task.

Finally, it is also possible that the lack of statistically significant transfer effects is partly due to the age group studied, as many executive functions have been shown to be at their peak in young adults [for set shifting], [ see for example 52–53]. Therefore, it may be that children and older adults would show greater training gains, as was the case in the Karbach and Kray [26] study.

The present study employed a no-contact control group design that has recently been criticized for potential confounds due to for example differences in effort, expectancy, and investment [4], [54]. However, despite these potential confounds, our results did not show any differences on the transfer tasks in favor of the training groups.

In summary, the results from the present study show that training set shifting with the categorization task, be it cued or uncued, fails to generate any robust transfer effects to other untrained tasks. In future studies, more attention needs to be paid to training-related features that can support generalization of learning, such as optimal levels of task difficulty, motivation and arousal of the participant, type of feedback given, and variability of the task [1].
